# Ferritin heavy subunit enhances apoptosis of non-small cell lung cancer cells through modulation of miR-125b/p53 axis

**DOI:** 10.1038/s41419-018-1216-3

**Published:** 2018-12-05

**Authors:** Flavia Biamonte, Anna Martina Battaglia, Fabiana Zolea, Duarte Mendes Oliveira, Ilenia Aversa, Gianluca Santamaria, Emilia Dora Giovannone, Gaetano Rocco, Giuseppe Viglietto, Francesco Costanzo

**Affiliations:** 1 Research Center of Biochemistry and Advanced Molecular Biology, Department of Experimental and Clinical Medicine, “Magna Græcia” University of Catanzaro, Campus Salvatore Venuta -Viale Europa, 88100 Catanzaro, Italy; 2 Department of Experimental and Clinical Medicine, University Magna Graecia of Catanzaro, Campus Salvatore Venuta -Viale Europa, 88100 Catanzaro, Italy; 3 Interdepartmental Center of Services (CIS), University Magna Graecia of Catanzaro, Campus Salvatore Venuta -Viale Europa, 88100 Catanzaro, Italy; 4 Department of Thoracic Surgical and Medical Oncology, Division of Thoracic Surgery, Istituto Nazionale Tumori, IRCCS, Pascale Foundation, Naples, Italy

## Abstract

Ferritin is a nanocage protein composed by the variable assembly of 24 heavy and light subunits. As major intracellular iron storage protein, ferritin has been studied for many years in the context of iron metabolism. However, recent evidences have highlighted its role, in particular that of the heavy subunit (FHC), in pathways related to cancer development and progression, such as cell proliferation, growth suppressor evasion, cell death inhibition, and angiogenesis. At least partly, the involvement in these pathways is due to the ability of FHC to control the expression of a repertoire of oncogenes and oncomiRNAs. Moreover, the existence of a feedback loop between FHC and the tumor suppressor p53 has been demonstrated in different cell types. Here, we show that ectopic over-expression of FHC induces the promoter hypermethylation and the down-regulation of miR-125b that, in turn, enhances p53 protein expression in non-small cell lung cancer (NSCLC) cell lines. Notably, analysis by absolute quantitative RT-PCR of *FHC*, *miR-125b,* and *p53* strongly suggests that this axis might be active in human NSCLC tissue specimens. In vitro, FHC over-expression attenuates survival of NSCLC cells by inducing p53-mediated intrinsic apoptosis that is partially abrogated upon miR-125b re-expression. Overall, our findings demonstrate that FHC acts as a tumor suppressor gene, thus providing a potential molecular strategy for induction of NSCLC apoptotic cell death.

## Introduction

Lung cancer is the leading cause of cancer mortality worldwide. It is a complex and heterogeneous group of diseases among which the non-small cell lung carcinoma (NSCLC) accounts for approximately 75–85% of all cases^1,2^. The complex molecular pathogenesis of NSCLC involves the activation of growth-promoting proteins (KRAS, MEK-1, EGFR, BRAF, etc.) as well as the inhibition of tumor suppressor genes (*p53*, *PTEN*, etc*.*)^3,4^. Particularly, the tumor suppressor protein p53 is frequently deleted or mutated in a large number of lung cancer cell lines thus leading to the deregulation of its downstream pathways^4^.

As recently described, p53 activities that impact on tumor suppression extend far beyond its role in promoting cell cycle arrest or apoptosis and include also the capacity to modulate glucose and lipid metabolism as well as the redox status of the cell^5–8^; consequently, p53 is now considered a central hub of multiple biochemical pathways essential for the cellular homeostasis and survival. As there are many pathways downstream of p53, so they are those upstream, being the tumor suppressor activated by a variety of stress signals including irradiation, DNA damage, oncogene expression, nutrient deprivation, and hypoxia^9,10^.

When the cell is exposed to damaging events, p53 activation is elicited by its stabilization, leading to increased intracellular amounts, and/or by the activation of the protein through a variety of post-translational modifications^11^. Recently, it has been reported that miRNAs are engaged in the activation pathway of p53 and its related networks at different levels through direct repression of the tumor suppressor or by the modulation of its regulators within the cell. miR-125b, miR-98, miR-150, miR-214, and miR-19b fall between the p53 direct modulators, since they directly seed-match sequences in the 3′-UTR of the tumor suppressor mRNA. On the contrary, miR-192/194/215, miR-143/145, miR-29b, miR-25, miR-32, and miR-18b indirectly affect p53 through the modulation of its regulators MDM2 and MDM4^12–14^.

p53 levels and/or activity are also affected by alterations of the intracellular redox state^15–17^. In response to cellular stress, an increased production of ROS induces p53 activation that, in turn, modulates the transcription of antioxidant target genes in order to prevent the propagation of the cell damage^15–17^. Ferritin plays a central role in the regulation of the intracellular redox homeostasis, due to its capacity to store iron atoms in a non-toxic form^18^. The variable assembly of 24 subunits of heavy (FHC) and light (FTL) types composes the protein shell provided of a central cavity in which iron is stored. The ferritin heavy subunit (FHC) plays a central role in this process since it is provided of a ferroxidase activity^19^. At the same time, data are mounting that FHC is involved in many pathways related to neoplastic transformation, as recently reviewed by Min Pang and Connor^20^; consequently, FHC is currently described as a bi-functional molecule involved in iron-related and iron unrelated biochemical routes. In many of these pathways p53 plays a central role and a tight relationship between p53 activity and FHC expression has been demonstrated^21–23^. p53 increases FHC expression at the translational level through the modulation of the IRP1-IRE translation regulatory system, while the over-expression of the tumor suppressor negatively modulates FHC transcription^22^. Conversely, FHC physically binds p53 and stabilizes the protein level under oxidative stress conditions^23^.

We have recently demonstrated that the intracellular increase of FHC amounts leads to enhancement of p53 protein expression in H460 non-small-cell lung cancer (NSCLC) cells and that this is accompanied by a significant reduction of the cell proliferation rate^24^. However, whether this FHC function is cell-specific or more ubiquitously effective as well as whether this function is directly mediated by perturbation of the redox metabolism or by iron-independent mechanisms are still to be explored.

In the present study we demonstrate the existence of a new regulatory axis through which FHC, regardless of its redox-related activity, enhances p53 expression by down-regulation of miR-125b steady-state amounts in A549, H460, SW1573, and LXF-289 NSCLC cells lines. The model has been also validated in human clinical specimens from NSCLC patients. Moreover, we found that FHC over-expression triggered a p53-dependent apoptotic cell death in vitro.

## Results

### miR-125b regulates p53 expression and activity in A549 and H460 cell lines

**Fig. 1:**
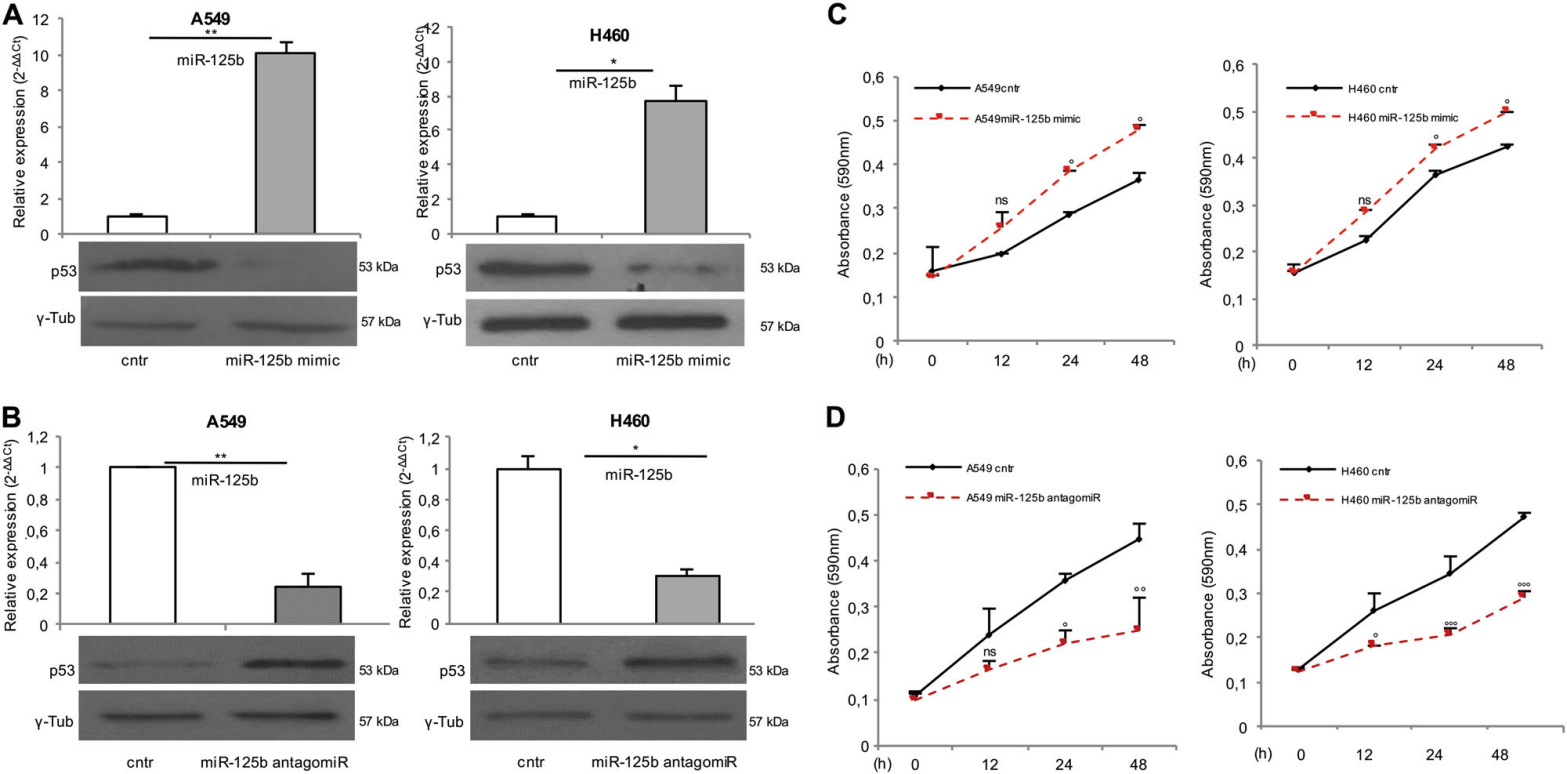
miR-125b negatively regulates p53 expression and cell growth in NSCLC cells. Taqman analysis of miR-125b expression in A549 and H460 cells upon 48 h transient transfection with miR-125b mimic (up) and relative p53 protein levels measured through Western Blot assay (down).*P < 0.05, **P < 0.01 (**a**). Taqman analysis of miR-125b expression in A549 and H460 cells upon 48 h transient transfection with miR-125b antagomiR (up) and relative p53 protein levels measured through Western Blot assay (down) *P < 0.05, **P < 0.01 (**b**). MTT assay of A549 (left) and H460 (right) cell lines at 12, 24, and 48 h upon miR-125b mimic transfection. ANOVA: °*P* < 0.05, ns: not significant. Each assay was performed in triplicate (**c**). MTT assay of A549 (left) and H460 (right) cell lines at 12, 24, and 48 h upon miR-125b antagomiR transfection. ANOVA:°*P* < 0.05, °°*P* < 0.01, °°°*P* < 0.001, ns: not significant. Each assay was performed in triplicate (**d**)

miR-125b negatively regulates p53 protein expression in human neuroblastoma and lung fibroblast cells^25^. To obtain experimental evidences supporting p53 as a target for miR-125b also in NSCLC cells, A549 and H460 cells were transfected with either a miR-125b mimic or antagomiR. After 48 h the expression of p53 protein was analyzed by western blot. The results shown in Fig. 1, representative of four independent transfections, demonstrated that miR-125b mimic caused at least 70% decrease of p53 protein in both cell lines (*P* < 0.05; Fig. 1a); by contrast, treatment with miR-125b antagomiR resulted in at least two-fold increase in p53 protein (*P* < 0.05; Fig. 1b). These experiments confirmed a suppressive role for miR-125b on the tumor suppressor p53 expression also in NSCLC cells. Next, we determined the effects of either miR-125b over-expression or inhibition on A549 and H460 cell viability. As shown in Fig. 1c both A549 and H460 cells transfected with miR-125b mimic (A549^miR-125b mimic^ and H460^miR-125b mimic^) showed an increase in cell growth compared with the A549^cntr^ and H460^cntr^ control cells at 24 and 48 h. On the other hand, A549 and H460 cells treated with miR-125b antagomiR (A549^miR-125b antagomiR^ and H460^miR-125b antagomiR^) exhibited decreased cell viability when compared with cells transfected with control siRNAs (A549^cntr^ and H460^cntr^) (Fig. 1d).

### FHC epigenetically regulates miR-125b in NSCLC cells

**Fig. 2:**
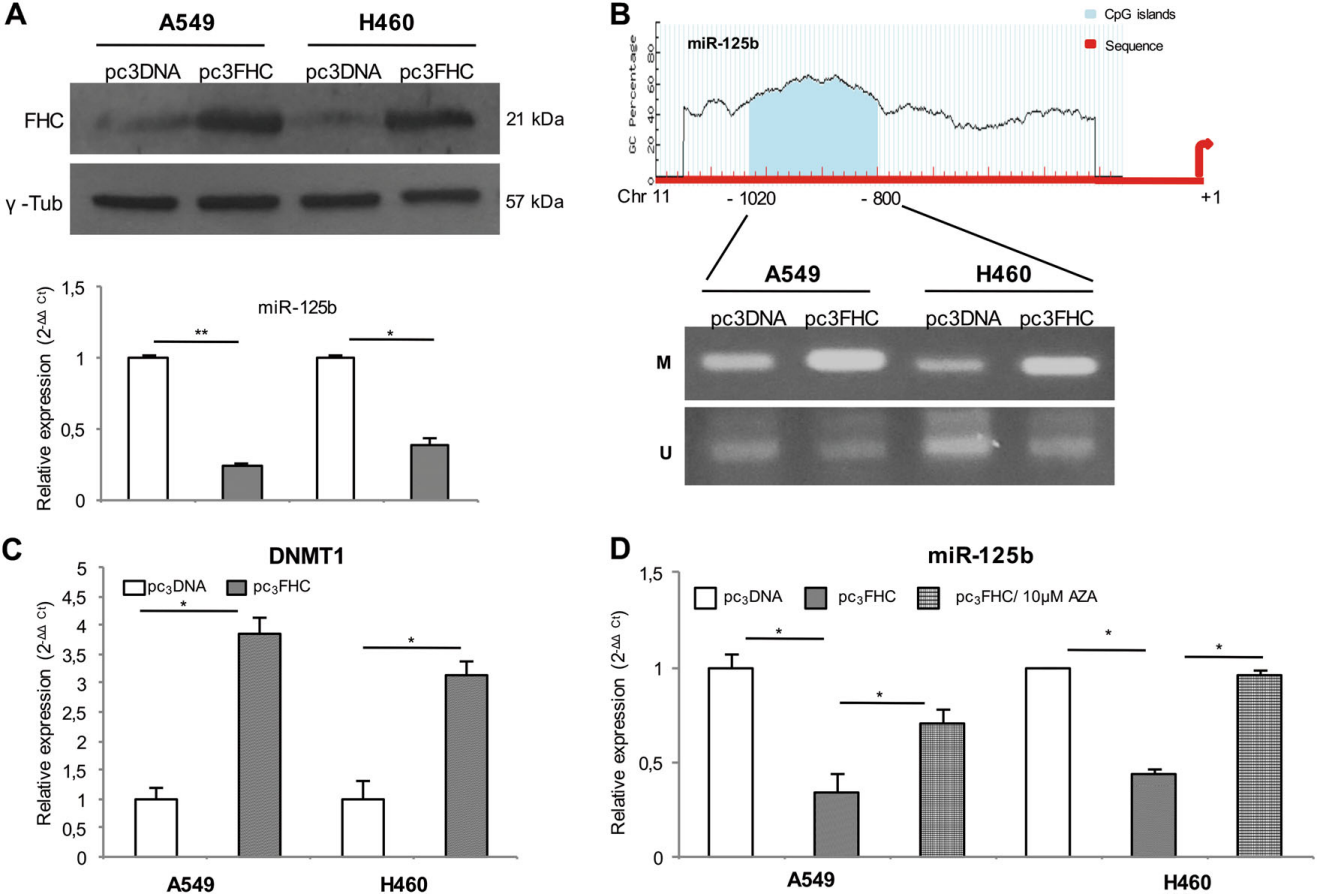
FHC suppresses miR-125b expression through the hypermethylation of its promoter in NSCLC cells. Representative western blot analysis of FHC protein levels in A549 and H460 cells transfected with either the control pc_3_DNA expression vector (A549^pc3DNA^ and H460^pc3DNA^) or the FHC-specific pc_3_FHC expression vector (A549^pc3FHC^ and H460^pc3FHC^) for 48 h (up). Taqman analysis of miR-125b expression in A549^pc3DNA^, A549^pc3FHC^, H460^pc3DNA^, and H460^pc3FHC^ cells (down). Each experiment was performed on three independent biological replicates. *P < 0.05, **P < 0.01 (**a**). Analysis of methylation status of a CpG site within the *miR-125b* gene promoter (up) through methylation-specific PCR (MSP) in A549^pc3DNA^, A549^pc3FHC^, H460^pc3DNA^, and H460^pc3FHC^cells (down). Representative image of three independent biological replicates. M methylated, U unmethylated (**b**). Real-time PCR analysis of *DNMT1* expression in A549^pc3DNA^, A549^pc3FHC^, H460^pc3DNA^, and H460^pc3FHC^cells; the *DNMT1* expression level was significantly enhanced in A549^pc3FHC^ and H460^pc3FHC^ cells compared to their relative controls A549^pc3DNA^ and H460^pc3DNA^ cells. The assay was performed in triplicate. *P < 0.05 (**c**). Taqman analysis of miR-125b expression in A549 and H460 control cells, and A549^pc3FHC^ and H460^pc3FHC^cells upon treatment with 10 µM 5-aza-2 deoxycytidine (AZA) for 48 h. The assay was performed in triplicate. *P < 0.05 (**d**)

FHC, beside its well-described role in the control of redox balance, exerts its cancer-related functions also modulating miR-125b expression in K562 and SKOV-3 cell lines^26,27^. Here, we analyzed the effects of transient FHC over-expression on miR-125b steady-state amounts in NSCLC cells (A549^pc3FHC^ and H460^pc3FHC^). Results of three independent experiments showed that ectopic over-expression of FHC significantly down-regulated miR-125b of at least 60% in both cell lines (*P* < 0.05; Fig. 2a).

To have an insight on FHC-mediated miR-125b regulation, we took into account that miR-125b expression is finely controlled by either an oxidative stress- or an epigenetically-dependent mechanism^28–30^. Thus, we measured ROS amounts in A549^pc3FHC^ and H460^pc3FHC^ cells compared to A549^pc3DNA^ and H460^pc3DNA^ control cells (Fig. S1). The absence of any significant alteration in ROS production let us to hypothesize that FHC regulated miR-125b expression through an iron/redox-independent mechanism. Next, we performed methylation-specific PCR (MSP) analysis of selected CpG islands identified within a −800 to −1020 promoter region upstream of miR-125b^30^. According to miR-125b low expression levels, this region was found to be hypermethylated in both A549^pc3FHC^ and H460^pc3FHC^ cells with respect to the relative control cells (Fig. 2b). A consistent up-regulation of DNA methyltransferase 1 (*DNMT1*) gene (*P* < 0.05; Fig. 2c) in A549^pc3FHC^ and H460^pc3FHC^ cells further supported this finding. Finally, the treatment of A549^pc3FHC^ and H460^pc3FHC^ cells with 10 µM of the demethylating agent decitabine (5‐aza‐2 deoxycytidine; AZA) for 48 h induced the up-regulation of miR-125b in both the cell lines (*P* < 0.05; Fig. 2d).

### FHC enhances p53 expression through the modulation of miR-125b in NSCLC cells

**Fig. 3:**
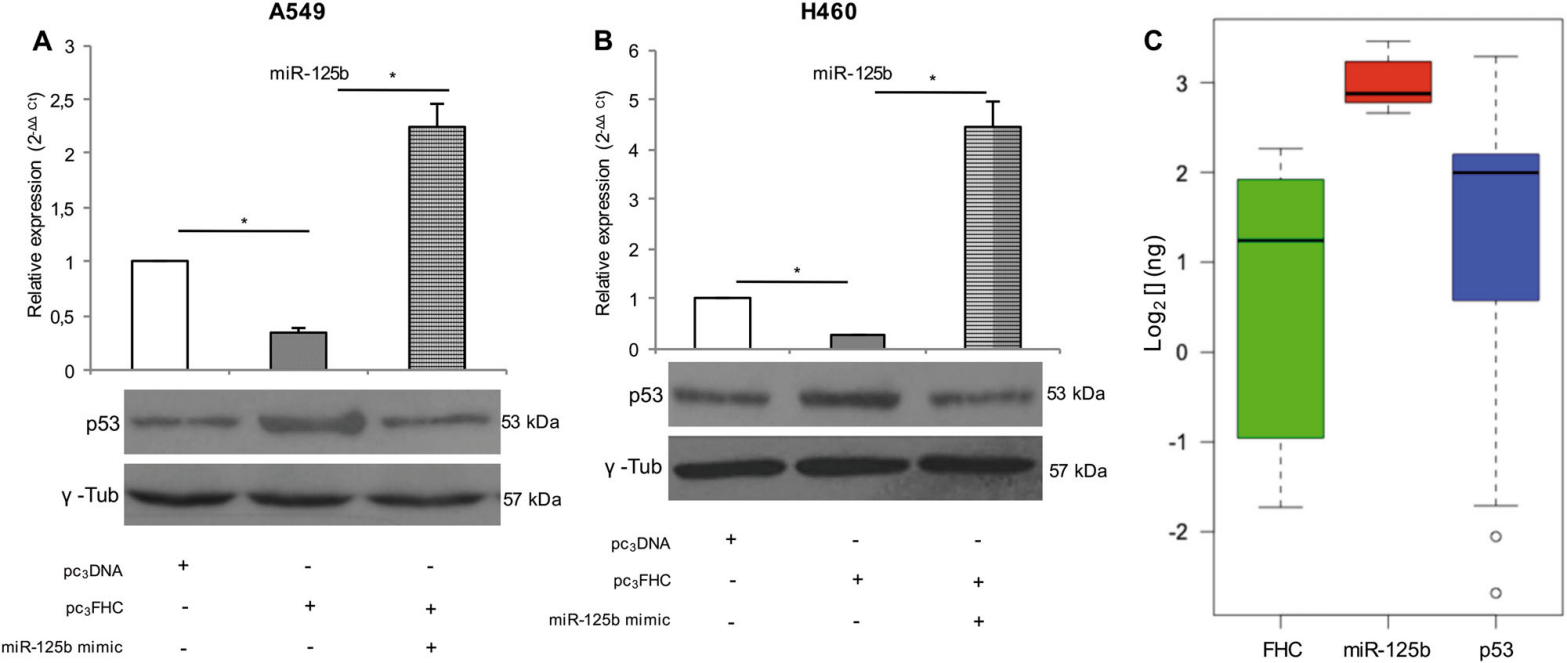
FHC enhances p53 expression through the negative modulation of miR-125b in NSCLC in vitro and ex vivo. Taqman analysis of miR-125b expression in A549 and H460 cells either transfected with pc_3_FHC expression vector alone (A549^pc3FHC^ and H460^pc3FHC^) or co-transfected with pc_3_FHC/miR-125b mimic (A549^pc3FHC/miR-125b mimic^ and H460^pc3FHC/miR-125b mimic^) (up). Western Blot assay revealed that protein p53 levels increased in A549^pc3FHC^ and H460^pc3FHC^ vs A549^pc3DNA^ and H460^pc3DNA^ control cells and decreased in A549^pc3FHC/miR-125b mimic^ and H460^pc3FHC/miR-125b mimic^ vs A549^pc3FHC^ and H460^pc3FHC^ (down). Each assay was performed on three independent biological replicates. *P < 0.05 (**a**, **b**). Absolute quantitative real-time PCR of *miR-125b*, *p53*, and *FHC* in 22 human NSCLC tissue specimens are reported as Box Plot. Absolute qPCR was performed on three independent technical replicates. Wilcoxon signed-rank test ***P < 0.001 *p53* vs *miR-125b*, Wilcoxon signed-rank test °°°*P* < 0.001 *FHC* vs *miR-125b* (**c**)

The above results indicate that, in A549 and H460 cells, miR-125b suppresses p53 protein expression and that FHC over-expression inhibits miR-125b. This suggests a new regulatory activity of FHC on p53 through the down-regulation of miR-125b. To verify this model, western blot analyses of p53 were performed in the cells transfected with the pc_3_FHC expression vector or co-transfected with pc_3_FHC and miR-125b mimic. Each transfection was performed at least three times. In the FHC-overexpressing NSCLC cells (A549^pc3FHC^ and H460^pc3FHC^) we observed a down-regulation of miR-125b accompanied by a roughly two-fold increase of p53 protein expression; these effects were counteracted when miR-125b levels were restored in the A549 and H460 cells co-transfected with pc_3_FHC and miR-125b mimic (A549^pc3FHC/miR-125b mimic^ and H460^pc3FHC/miR-125b mimic^) (*P* < 0.05; Fig. 3a, b).

Moreover, the FHC/miR-125b/p53 axis was further analyzed in 22 human tumor tissue specimens derived from NSCLC patients (patient characteristics are reported in Table 1) by quantifying the three molecules transcript amounts through absolute qPCR. As represented in the box plot in Fig. 3c, non-parametric Wilcoxon signed-rank test revealed a very significant negative correlation between the expression of p53 and miR-125b (*P* = 4.503e-09) and between FHC and miR-125b (*P* = 3.716e-12). Instead, the Wilcoxon signed-rank test returned a not significant *p*-value between p53 and FHC (Fig. 3c).

**Table 1:** Patient characteristics

	Frequency (number)
**Age at diagnosis (years)**	
<50	5% (1)
50–59	14% (3)
60–69	31% (7)
>70	50% (11)
**Histotype**	
Adenocarcinoma	32% (7)
Squamous cell carcinoma	23% (5)
Large cell carcinoma	9% (2)
Not otherwise specified (NOS)	36% (8)
**Grade**	
I	0
II	55% (12)
III/IV	31% (7)
Unknown	14% (3)
**Staging**	
I	36% (8)
II	9% (2)
III	5% (1)
IV	41% (9)
Unknown	9% (2)

Collectively, these results support a so far uncovered model in which FHC regulates p53 through its negative control on miR-125b

### FHC/miR-125b/p53 axis affects A549 and H460 cell survival by modulating the intrinsic apoptotic pathway

**Fig. 4:**
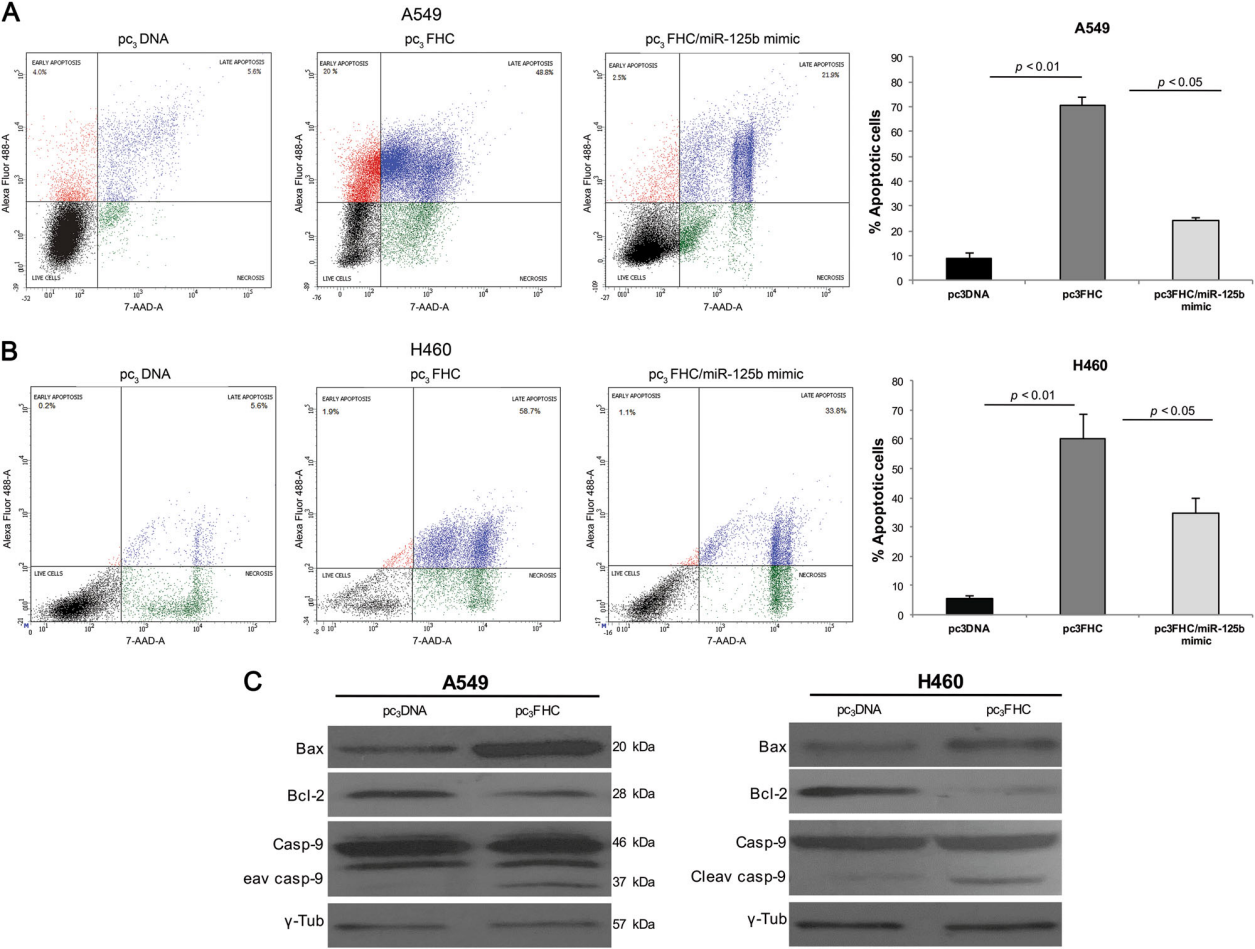
FHC over-expression promotes intrinsic apoptosis in NSCLC cells. Representative plots of Annexin V/7-AAD apoptosis assays in A549^pc3DNA^, A549^pc3FHC^, A549^pc3FHC/miR-125b mimic^ (left), and graphical data of total apoptotic cells (%) in each sample (right) (**a**). Representative plots of Annexin V/7-AAD apoptosis assays in H460^pc3DNA^, H460^pc3FHC^, H460^pc3FHC/miR-125b mimic^ (left) and graphical data of total apoptotic cells (%) in each sample (right) (**b**). FACS analysis was performed on three independent biological replicates. Western blot analysis of three independent biological replicates of BAX, Bcl2, Caspase-9, and cleaved Caspase-9 protein levels in A549^pc3DNA^, A549^pc3FHC^ and H460^pc3DNA^, H460^pc3FHC^. *P < 0.05 (**c**)

To assess how the FHC/miR-125b/p53 axis impacted on NSCLC cell behavior, we carried out the cytofluorimetric cell cycle and apoptosis assays. Representative graphs from PI flow cytometry analyses demonstrate that FHC ectopic over‐expression did not alter A549 and H460 cell distribution within the major phases of the cell cycle (Fig. S2). Conversely, representative plots and quantifications of Annexin V/7-AAD apoptosis assays highlighted that the percentage of apoptosis, considered as the sum of early apoptosis (Annexin V^+^/7-AAD^−^) and late apoptosis (Annexin V^+^/7-AAD^+^), was remarkably increased in A549 and H460 FHC-overexpressing cells compared with the relative controls (A549^pc3FHC^ vs A549^pc3DNA^: 70.6% ± 3.4 vs 9.1% ± 1.9, *P* < 0.01) (H460^pc3FHC^ vs H460^pc3DNA^: 60.2% ± 8.4 vs 5.8% ± 0.8, *P* < 0.01) (Fig. 4a, b). In addition, the mimic-mediated miR-125b rescue in A549^pc3FHC/miR-125b mimic^ and H460^pc3FHC/miR-125b mimic^ was able to partially counteract the pro-apoptotic effect of the sole H-ferritin over-expression (A549^pc3FHC/miR-125b mimic^: 24.2% ± 1.05, *P* < 0.05) (H460^pc3FHC/miR-125b mimic^: 34.9% ± 5.1, *P* < 0.05) (Fig. 4a, b).

Moreover, western blot analyses showed an increase in BAX/Bcl-2 ratio, indicative of the activation of the intrinsic apoptotic pathway within A549^pc3FHC^ and H460^pc3FHC^ cells, further confirmed by the increased caspase-9 cleavage (Fig. 4c). Otherwise, western blot analysis of FAS and caspase-8 cleavage suggested that extrinsic apoptotic pathway was not involved (Fig. S3).

### FHC/miR-125b/p53 axis modulates apoptosis in SW1573 and LXF-289 NSCLC cells

To evaluate the generalizability of FHC/miR-125b/p53 axis in NSCLC cells, the amounts of miR-125b and p53 were determined in SW1573 and LXF-289 cells upon FHC over-expression. Results confirmed that the increase in FHC amounts is accompanied by miR-125b down-regulation that, in turn, enhances p53 levels in both the cell lines (Fig. S4a). We further explored the functional effects of p53 modulation on the apoptotic rate of both SW1573 and LXF-289 cells. As reported in the representative plots in Fig. S4b, the quantification of Annexin V/PI-positive cells highlighted that FHC over-expression increased the percentage of apoptosis, considered again as the sum of both early apoptosis (Annexin V^+^/PI^−^) and late apoptosis (Annexin V^+^/PI^+^): SW1573^pc3FHC^ vs SW1573^pc3DNA^ : 35.1% ± 2.1 vs 14.1% ± 0.3, *P* < 0.05) (LXF-289^pc3FHC^ vs LXF-289^pc3DNA^: 15.0% ± 0.4 vs 5.1% ± 0.3, *P* < 0.05).

## Discussion

Numerous evidences link intracellular iron metabolism and tumorigenesis^31,32^; among them, a segment puts directly in relation iron and tumor suppressor p53 functions. Indeed, it has been demonstrated that iron depletion up-regulates p53 at post-transcriptional level^33^, as well as that heme is able to bind p53 and to down-regulate its activity, by modifying localization and stability^34^. p53 is able, for its part, to modulate the intracellular iron homeostasis: this observation stems from the discovery that ISCU (iron–sulfur cluster assembly enzyme) belongs to the family of p53 target genes, being provided with an intronic p53-binding site. ISCU, in turn, controls, at post-transcriptional level, the expression of FHC and transferrin receptor (TFRC), two key proteins of iron metabolism; in particular, by acting on the IRP1/IRE regulatory system, *ISCU* positively modulates FHC translation and negatively modulates TFRC mRNA half-life^35^.

The links between p53 and FHC do not end with the ISCU post-transcriptional role: upon oxidative stress, FHC might physically interact with p53 and increase its transcriptional activity^23^. The interaction and the downstream p53 activation also takes place with a mutant form of FHC devoid of ferroxidase activity, strongly suggesting that the binding capability is iron-independent. On the other hand, p53, following its over-expression, is recruited by the NF-Y transcription factor onto the FHC promoter, where it acts as a strong negative regulator of ferritin transcriptional efficiency^22^. Finally, it has been shown that H-chain rich acidic isoferritins, released from hepatocytes in vitro, up-regulate p53 and mediate apoptotic processes through a mechanism which again appears to be unrelated to the ferritin role in balancing the intracellular iron pool^36^.

**Fig. 5:**
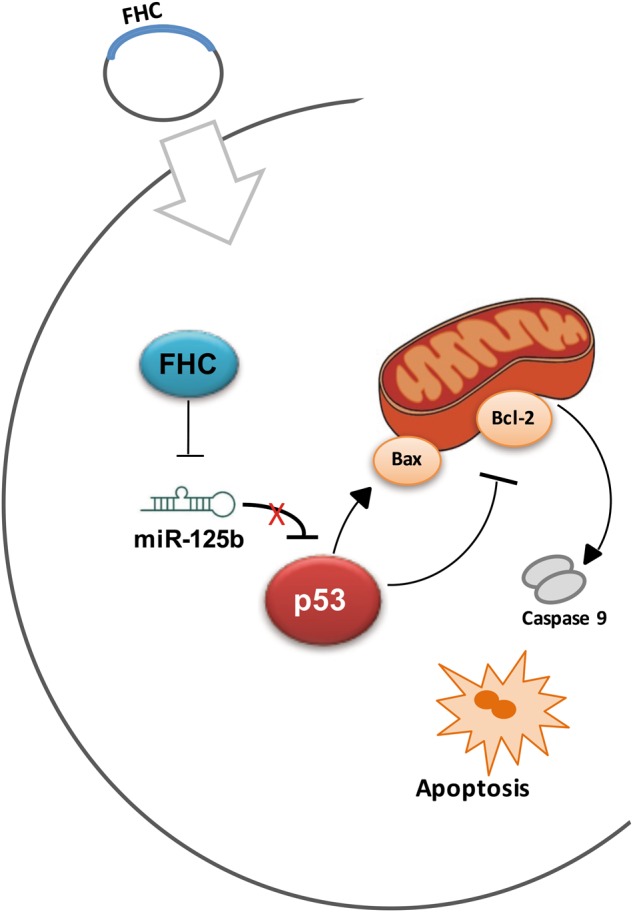
Model of the FHC/miR-125b/p53 axis in NSCLC. Over-expression of FHC (blue circle) leads to the repression of microRNA 125b. miR-125b inhibition promotes the translation of p53 (red circle) that, in turn, leads to the over-expression of pro-apoptotic protein BAX and the inhibition of the anti-apoptotic Bcl2. Consequently, disruption of the mitochondrial membrane potential (MMP) unleashes the enzymatic apoptotic machinery of caspases, and in particular caspase-9

The major finding of our work is the identification of a novel and so far uncovered relationship between FHC and p53 in NSCLC cells through miR-125b. The set of our results indicate that FHC is the upstream molecule of this regulatory axis by virtue of its ability to modulate miR-125b amounts; the microRNA, for its parts, controls the expression and therefore the activity of p53 (Fig. 5). Analyses of the molecular mechanisms underlying this regulatory axis strongly suggest that FHC exerts its effects on miR-125b/p53 regardless of the iron/redox status of the cell being ROS production unaltered upon FHC over-expression (Fig. S1).

On the contrary, several observations indicate that FHC epigenetically suppresses miR-125b expression: (i) FHC over-expression is accompanied by the hypermethylation of a previously computationally mapped CpG island in miR-125b promoter region^30^; (ii) FHC over-expression is accompanied by the up-regulation of *DNMT1* gene expression, and (iii) miR-125b expression is partially restored after treatment of FHC-overexpressing cells with the demethylating agent 5-Aza for 48 h. The observation that *DNMT1* steady-state amounts are induced by FHC over-expression deserves further and more detailed investigations. *DNMT1* has been described as a direct target of a repertoire of miRNAs including miR-126^37,38^. We already found that miR-126 belongs to the FHC-dependent miRNAs in K562 erythroleukemia cells^26^. Thus, we determined miR-126 levels in A549, H460, SW1573, LXF-289 control, and FHC-overexpressing cells. Results show that miR-126 is down-regulated, at variable extents, in the FHC-overexpressing cells (A549^pc3FHC^, H460^pc3FHC^, SW1573^pc3FHC^, and LXF-289 ^pc3FHC^) where *DNMT1* is up-regulated (data not shown).

p53 is a target of miR-125b in human neuroblastoma cells and in lung fibroblasts^25^; here, by using miR-125b mimic and antagomiR we demonstrate that this control also takes place in NSCLC cells. Consequently, miR-125b, through its suppressive activity on p53, acts as oncomiRNA in NSCLC cells; accordingly, cells transfected with miR-125b antagomiR show a consistent growth decrease. Moreover, the miRNA intermediate position between FHC and p53 is confirmed by recovery experiments in which a specific mimic is used to restore miR-125b levels suppressed upon FHC over-expression. By absolute RT-qPCR analysis we also determined the steady-state amounts of *FHC*, *miR-125b,* and *p53* in 22 NCSLC tissue samples. Wilcoxon signed-rank test highlights that the levels of *FHC* and *miR-125b* are inversely related, as are those of miR-125b and p53, with a high statistical significance; this strongly suggests that the relationship of FHC with p53 through miR-125b might be also active in vivo. Further analyses on a large size of clinical samples as well as in mouse models are required to confirm these findings.

p53 activity as a tumor suppressor depends on its ability in regulating downstream pathways involved in DNA repair, senescence, cell-cycle arrest, and/or apoptosis. Depending on the extent of injury, p53 may promote cell cycle arrest to activate DNA repair mechanisms and restore homeostasis; conversely, in case of a severe injury p53 induces cells to undergo death by apoptosis^9,10,39,40^. In this work, we show that FHC/miR-125b-mediated up-regulation of p53 forces NSCLC cells to undergo apoptosis without affecting cell cycle progression, thus suggesting that the wealth of the ferritin subunit could constitute a severe damage to the cell. Apoptosis is driven by p53 transcriptional induction of a number of genes, including the death receptors Fas and Dr4, which in turn activate the extrinsic apoptotic pathway^9,41^. Furthermore, p53 may increase the transcription of death agonist genes such as BAX and, in parallel, repress the expression of death antagonist such as Bcl-2, thus inducing mitochondrial outer membrane permeabilization and the intrinsic apoptosis pathway^9,41^. Here, we show that FHC/miR-125b/p53-mediated apoptosis occurs through the activation of the intrinsic pathway as indicated by the increase of the Bax/Bcl-2 ratio and caspase-9 activation.

As recently reviewed, the role of FHC in malignancies is conflicting since the ferritin subunit might function as a tumor suppressor or oncogene depending on the cellular context^20^. The natural conclusion of our findings on NSCLC cells is that FHC acts as a tumor suppressor in these cells as it happens in breast and ovarian cancer cell lines^27,42^. This role is exerted by H-ferritin via multiple mechanisms such as the regulation of CXCL12/CXCR-4 axis in breast cancer cells or the modulation of a specific microRNA pattern in ovarian cancer cells^27,40^. Some of these mechanisms might be also active in NSCLC cells along with the predominant modulation of p53. This is suggested by the observation that a percentage of apoptotic cells still remains when the miR-125b/p53 axis is contrasted by miR-125b mimic co-transfection.

Finally, the ability of FHC over-expression in triggering p53-mediated apoptosis might have profound implications in designing new therapeutic approaches in NSCLC, also taking into account its current employment as a nanocarrier due to its unique protein architecture.

## Materials and methods

### Cell cultures, human tissue samples, and treatments

Human NSCLC cell lines A549, H460, SW1573, and LXF-289 were obtained from the American Type Culture Collection (ATCC, Rockville, MD, USA). All the cell lines were maintained in RPMI1640 media (Sigma-Aldrich, St. Louis, MO, USA) supplemented with 10% (v/v) fetal bovine serum (FBS) (Invitrogen, San Diego, CA) and 1% (v/v) penicillin and streptomycin (Sigma-Aldrich, St. Louis, MO, USA), in adherent cultures at 37 °C in a humidified 5% CO_2_ atmosphere. Cell lines were tested for mycoplasma contaminations and STR profiled for authentication.

Twenty-two tumor biopsies were obtained from the University Magna Graecia of Catanzaro (Catanzaro, Italy); patient accrual was conducted according to the Internal Review Board of the INT Fondazione Pascale (Naples, Italy) (CEI:556,10 of 12/3/2010). The study was approved by the Internal Review Board of Mater Domini/University Magna Graecia (Catanzaro, Italy), in the meeting of 16/03/2011.

Twenty-two cases of NSCLC patient specimens were frozen and stored in liquid nitrogen until use. The characteristics of patients included are described in Table 1.

For 5-aza-2′-deoxycytidine (AZA) treatment, A549^pc3FHC^, and H460^pc3FHC^ were seeded at 5 × 10^5^ per well in six-well plates and cultured with 10 µM AZA (Sigma-Aldrich) for 48 h, respectively. The medium containing drug was replaced every 24 h. RNA was isolated and qRT-PCR was carried out to evaluate the restoration of miR-125b after AZA treatment.

### In vitro transfection of A549 and H460 cells with synthetic miR-125b antagomiR or mimic

Synthetic miR-125b antagomiR or mimic were purchased from Thermo Fisher Scientific (Waltham, Massachusetts, USA). A549 and H460 control cells (5 × 10^5^) were seeded in a six-well plate (Corning Incorporated, Corning, NY, USA). After 24 h, cells were transfected with 50 nM of hsa-miR-125b-5p mirVana miRNA mimic (A549^miR-125b mimic^ and H460^miR-125b mimic^) or 50 nM of has-miR-125b-5p mirVana miRNA antagomiR (A549^miR-125b antagomiR^ and H460^miR-125b antagomiR^) using Lipofectamine 2000 reagents according to the manufacturer's instructions (Thermo Fisher Scientific, Waltham, Massachusetts, USA). Fifty nanomolar of mirVana mimic or antagomiR-negative controls were also used. After 6 h of incubation, the transfection medium was replaced with fresh RPMI1640 medium. Then, after 48 h of transfection, cells were centrifuged and total RNA was extracted from A549^miR-125b mimic^ and H460^miR-125b mimic^, A549^miR-125b antagomiR^ and H460^miR-125b antagomiR^, A549^cntr^ and H460^cntr^.

### FHC transient over-expression in NSCLC cells

A549, H460, SW1573, and LXF-289 cells were plated into six-well plates at 5 × 10^5^ cells/well and grown overnight prior to transfection. *FHC* transient over-expression was performed by using a specific pc_3_FHC expression vector (A549^pc3FHC^, H460^pc3FHC^, SW1573^pc3FHC^ and LXF-289^pc3FHC^) and pc_3_DNA as negative control (A549^pc3DNA^, H460^pc3DNA^, SW1573^pc3DNA^, and LXF-289^pc3DNA^) as previously reported in Zolea et al.^24^. All transfections were performed three times using the Lipofectamine 2000 reagents according to the manufacturer's recommendations (Thermo Fisher Scientific, Waltham, Massachusetts, USA). After 48 h, FHC-specific over-expression was checked at protein levels through western blot. Data concerning FHC over-expression are reported as mean of three independent biological replicates.

### RNA isolation and qRT-PCR analysis

Total RNA isolation, single-stranded complementary DNA (cDNA) generation and relative-quantitative RT-PCR were performed as previously reported in Zolea et al.^24^. Validated qRT-PCR primers for *DNMT1* were from Thermo Fisher Scientific. For miRNA quantification we used a TaqMan MicroRNA assay kit (Thermo Fisher Scientific, Waltham, Massachusetts, USA) and specific primer sets for U6 snRNA (Assay ID: 001973) and mature hsa-miR-125b-5p (Assay ID: 000449) (Thermo Fisher Scientific, Waltham, Massachusetts, USA) according to the manufacturer’s instructions. GAPDH and U6 snRNA were used as internal normalizers for mRNA and miRNA, respectively.

Absolute qRT-PCR was used to determine the expression of *FHC*, *p53,* and *miR-125b* in 22 tumor tissue specimens. Briefly, starting from a sample of known template concentration, a 5-point 10-fold serial standard curve was prepared, and the concentration of all other samples was calculated by simple interpolation of each threshold cycle (Ct) into this standard curve. Gene expression data are reported as log_2_ [] (ng) and represent the mean of three independent technical replicates.

### Western blotting

Total cell lysates were prepared using RIPA buffer^24,26^. Each protein sample (40–50 μg) was separated by 10–15% SDS–PAGE and then transferred to nitrocellulose membranes^24,26^. Membranes were incubated with primary antibodies at 4 °C overnight. Antibodies against FHC (B-12) (1:200, sc-376594), p53 (A-1) (1:500, sc-393031), BAX (B-9) (1:500, sc-7480), and Bcl-2 (100) (1:500, sc-509) were purchased from Santa Cruz Biotechnology (Santa Cruz Biotechnology, Dallas, Texas); antibodies against Caspase-9 (1:500, #9502), Caspase-8 (1C12) (1:500, #9746), and Fas (C18C12) (1:500, #4233) were purchased from Cell Signaling Technology (Danvers, Massachusetts, USA). Membranes were then incubated with secondary antibodies, HRP-conjugated goat anti-mouse IgG (1:2000, sc-516102; Santa Cruz Biotechnology, Dallas, Texas) and HRP-conjugated mouse anti-rabbit IgG (1:2000, sc-2357; Santa Cruz Biotechnology, Dallas, Texas), and immunoreactive bands were visualized with the ECL western blotting detection system (Santa Cruz Biotechnology, Dallas, Texas). To ensure equal loading of proteins a goat polyclonal anti-γ-Tubulin antibody (C-20) (1:3000; sc-7396; Santa Cruz Biotechnology) was used. The protein band intensity on western blots was quantified and normalized to that of γ-Tubulin by using ImageJ software (http://rsb.info.nih.gov/ij/).

### MTT assay

A total of 5 × 10^4^ cells/well were seeded into a 24-well plate. 3-[4,5-Dimethylthiaoly]-2,5-diphenyltetrazolium bromide (MTT) (Sigma Aldrich, St. Louis, MO, USA) assay was performed to detect proliferation of A549 and H460 upon transient over-expression of miR-125b (A549^miR-125b mimic^ and H460^miR-125b mimic^) or transient inhibition of miR-125b (A549^miR-125b antagomiR^ and H460^miR-125b antagomiR^) for 12, 24, and 48 h. Fresh MTT 2mg/ml (Sigma Aldrich, St. Louis, MO, USA), re-suspended in phosphate-buffered saline (PBS), was added to each well. After 2 h incubation, culture medium was discarded and replaced with 200 μl of isopropanol. Optical density was measured at 590 nm in a spectrophotometer. Each transfection was performed in triplicate. For each sample, MTT assay was performed in quadruplicate.

### ROS detection

ROS were determined by incubating A549^pc3DNA^, H460^pc3DNA^, A549^pc3FHC^, and H460^pc3FHC^ with the redox-sensitive probe 2′-7′-DCF (CM-H2DCFDA; Thermo Fisher Scientific, Waltham, Massachusetts, USA)^43^.

### Methylation-specific PCR

MSP for detecting the methylation status of miR-125b promoter was performed as previously reported by Zhang et al.^30^. Bisulfite conversion was performed using EpiTect Bisulfite Kit Specific (Qiagen, Hilden, Germany); PCR was performed using AmpliTaq Gold™ DNA Polymerase Kit and primers as reported by Zhang et al.^30^.

### Cell cycle analysis

A total of 2 × 10^6^ cells were fixed with 100% ethanol and stored at 4 °C for overnight. Cells were rehydrated with PBS for 10 min at RT and then cells were stained with propidium iodide (PI) staining solution contained with 50 μg/ml PI (Sigma Aldrich, St. Louis, MO, USA), 100 μg/ml DNase-free RNase A (Calbiochem, La Jolla, CA), and 0.01% NP-40 (USB, Cleveland, OH) in PBS for 60 min at room temperature. Stained cells were analyzed for cell cycle analysis in BD LSRFortessa^TM^X-20 (BD Biosciences, San Jose, CA) and FlowJo software.

### Apoptosis analysis

For identifying cells actively undergoing apoptosis, a double staining with Annexin V and PI was performed using Alexa Fluor®488 Annexin V/Dead Cell Apoptosis Kit (Thermo Fisher Scientific, Waltham, Massachusetts, USA) according to the manufacturer’s instructions. After staining, cells were incubated at room temperature for 15 min in the dark. Each tube was diluted with 400 µl of Annexin Binding Buffer and then cells were analyzed by flow cytometry using the BD LSR Fortessa^TM^X-20 (BD Biosciences, San Jose, CA) and FACS Diva 7.0 program (BD Biosciences, San Jose, CA).

### Statistics

Where appropriate, data were evaluated by performing a simple comparison between two values using Student’s *t*-test. With *t*-tests, we compared the mean of each group assuming that the groups consisted of independent observations with equal variances. When testing time series, the assumption of independence is usually not reasonable. Consequently, even comparing the means of two or more time series is considerably more difficult than with independent data. So, when we were interested in determining whether the means from more than two groups or time series were equal or not, we performed analysis of variance (ANOVA). Summary statistics are presented as the mean ± S.D. A *P*-value of <0.05 was considered statistically significant.

Wilcoxon signed-rank test was used to determine the statistical significance of genes expression across the lung cohort of patients measured by absolute qRT-PCR. Expression values were defined as log_2_. A *P*-value of <0.05 was considered statistically significant.

## Electronic supplementary material

Supplementary Figure 1

Supplementary Figure 2

Supplementary Figure 3

Supplementary Figure 4

Supplementary figure legends
