# Supplementary figures and images for: Ferritin heavy subunit enhances apoptosis of non-small cell lung cancer cells through modulation of miR-125b/p53 axis
Source: Cell Death Dis. 2018 Dec 5;9(12):1174. doi: 10.1038/s41419-018-1216-3 (PMC6281584; doi:10.1038/s41419-018-1216-3)

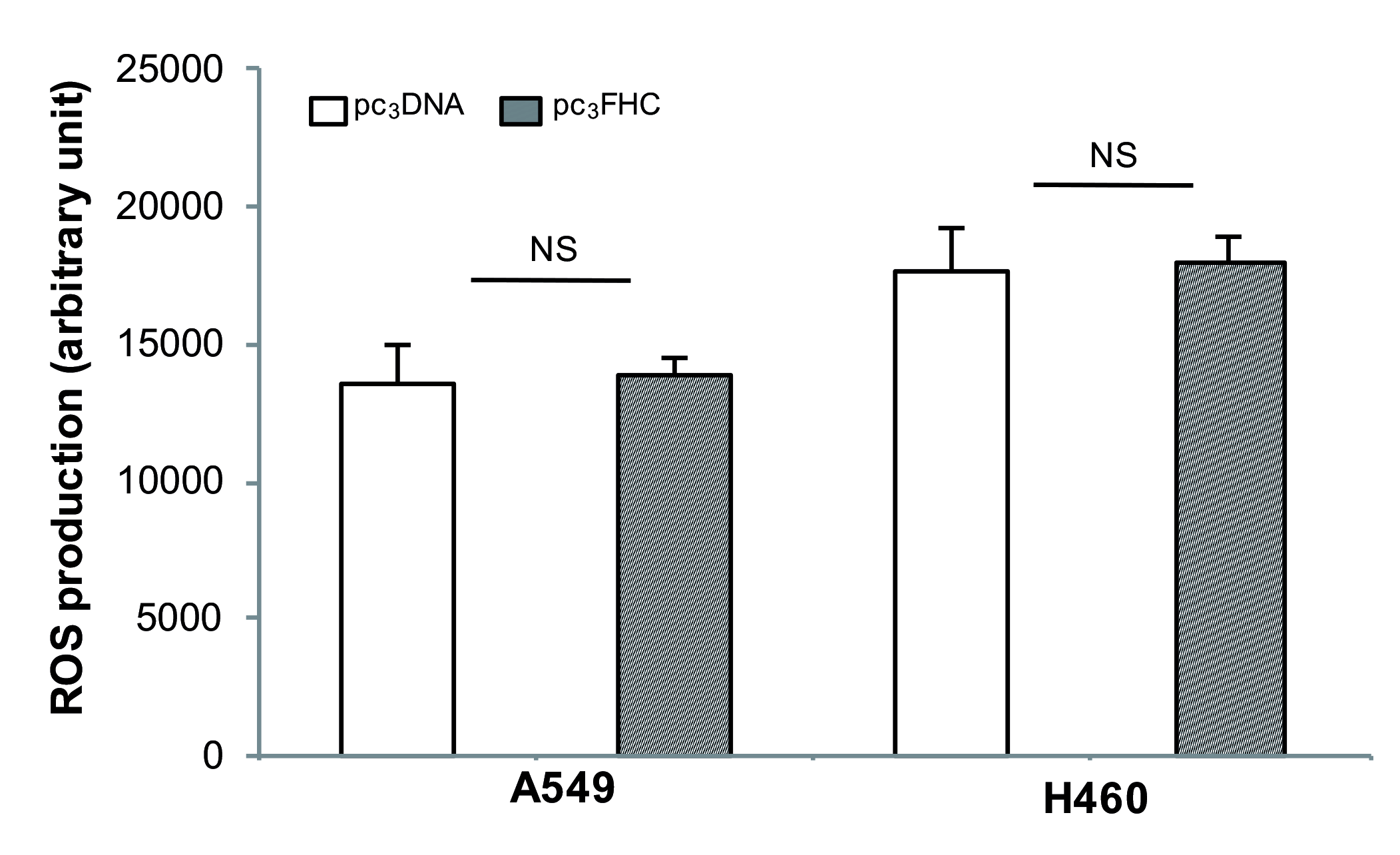

Supplement: Supplementary file 1 — Supplementary Figure 1 [file 41419_2018_1216_MOESM1_ESM.tif]

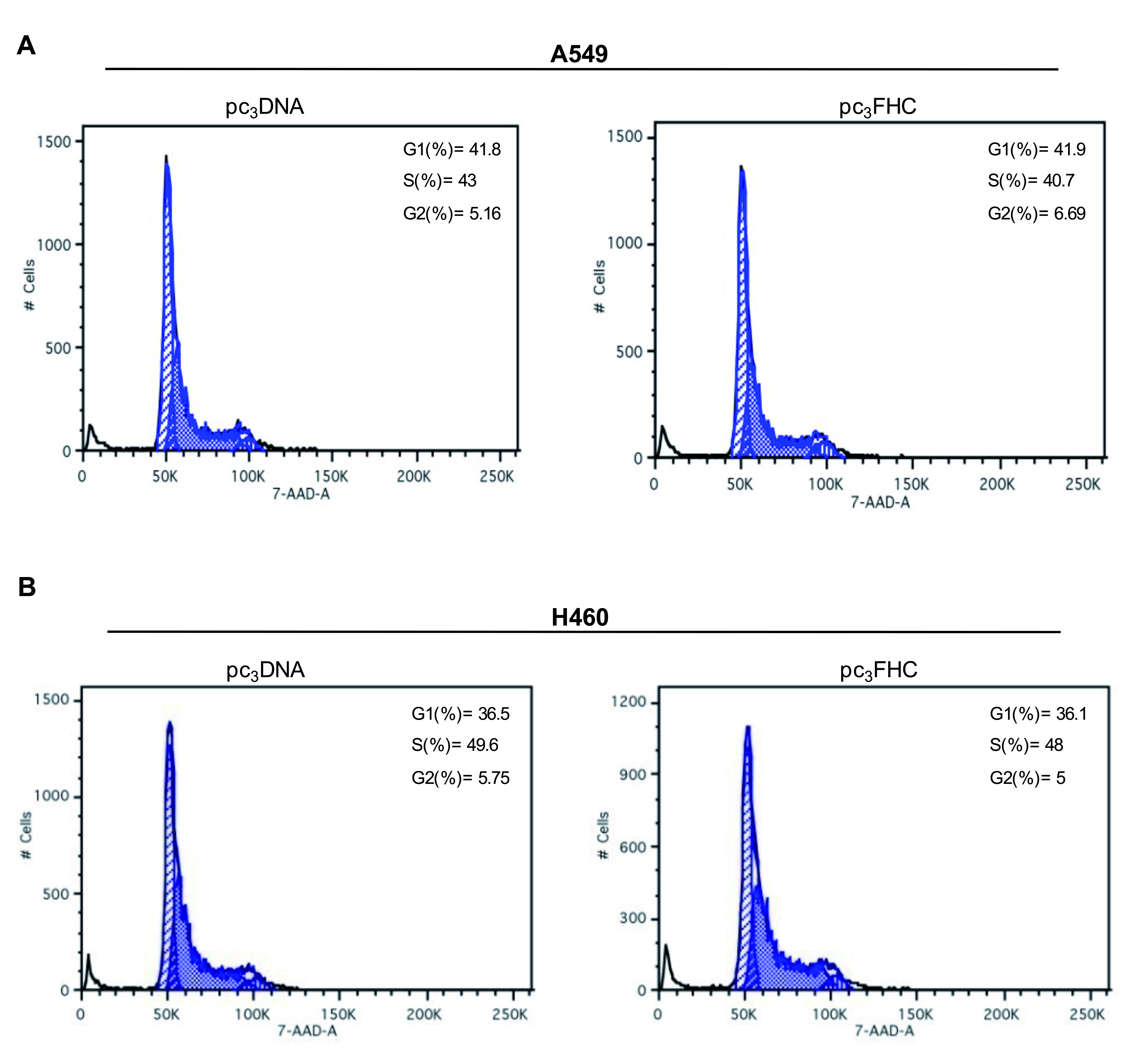

Supplement: Supplementary file 2 — Supplementary Figure 2 [file 41419_2018_1216_MOESM2_ESM.tif]

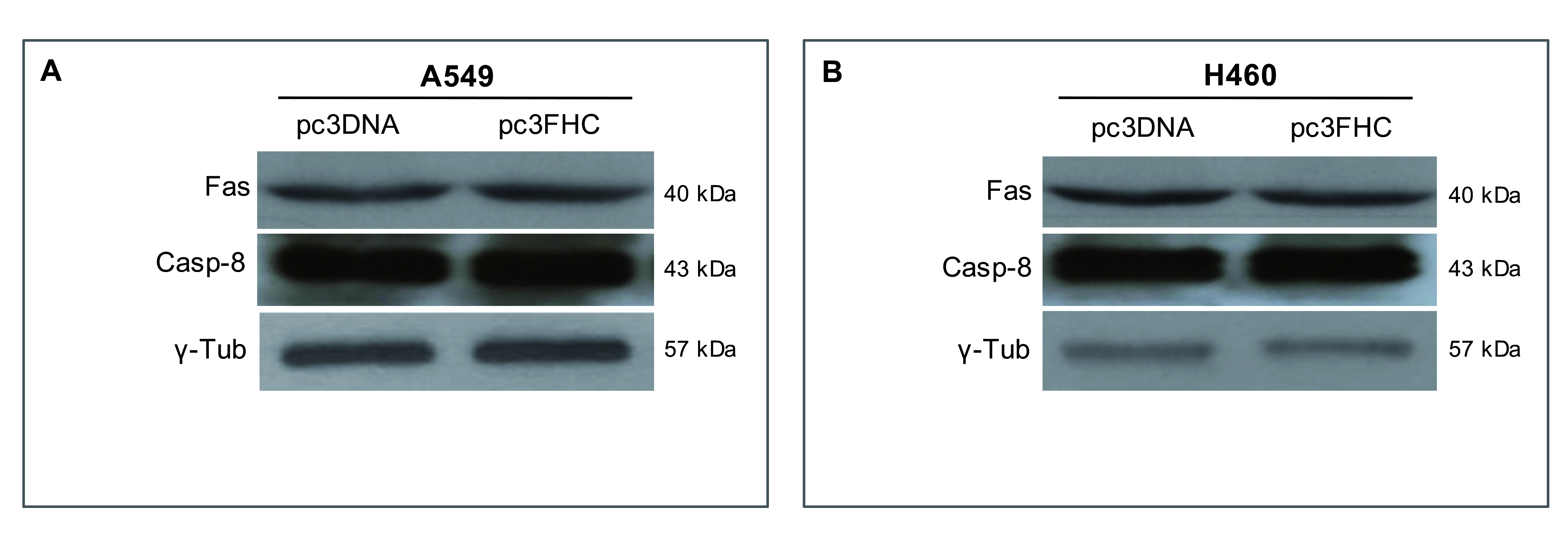

Supplement: Supplementary file 3 — Supplementary Figure 3 [file 41419_2018_1216_MOESM3_ESM.tif]

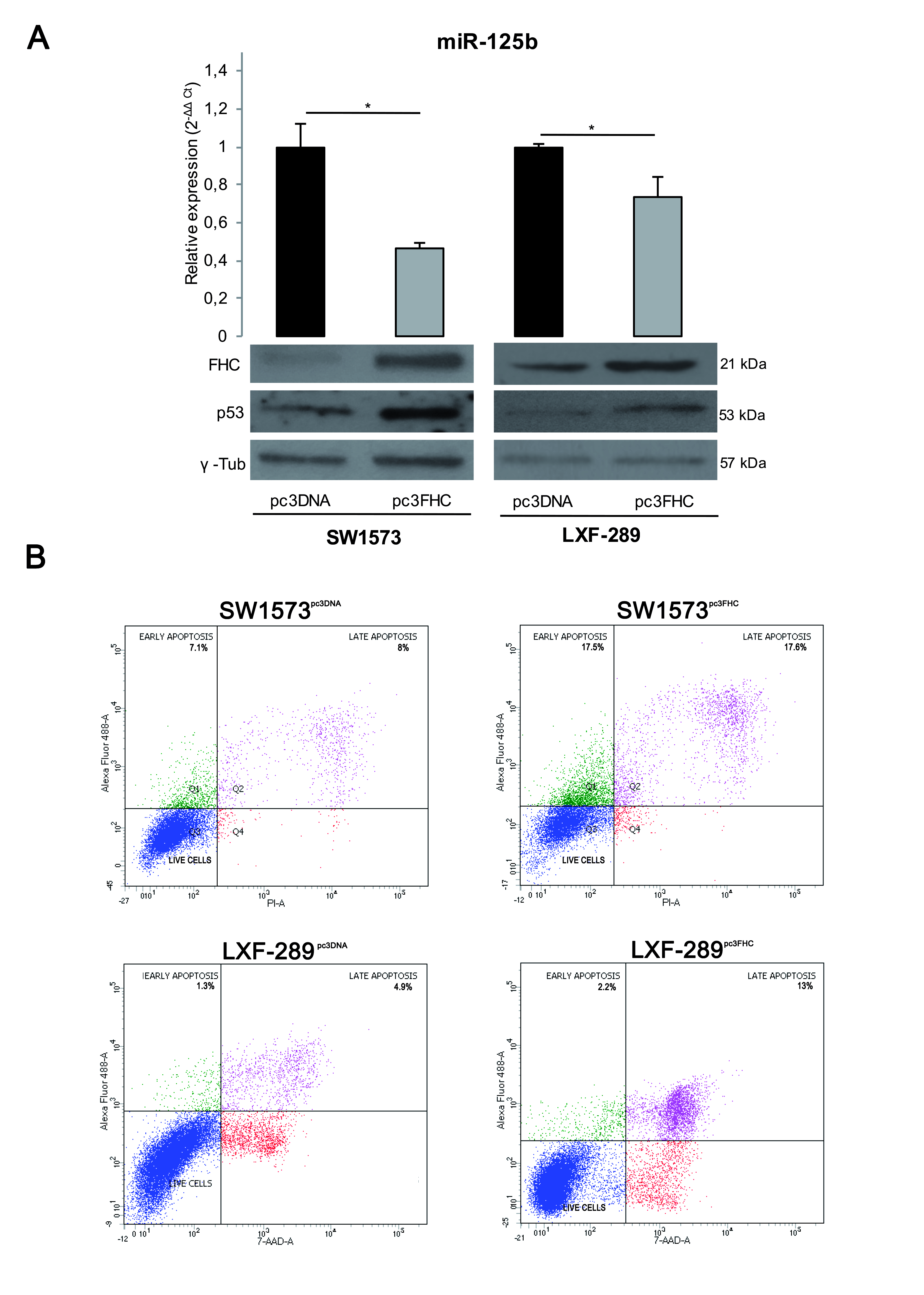

Supplement: Supplementary file 4 — Supplementary Figure 4 [file 41419_2018_1216_MOESM4_ESM.tif]
