# Supplementary material for: Ferritin heavy subunit enhances apoptosis of non-small cell lung cancer cells through modulation of miR-125b/p53 axis
Source: Cell Death Dis. 2018 Dec 5;9(12):1174. doi: 10.1038/s41419-018-1216-3 (PMC6281584; doi:10.1038/s41419-018-1216-3)
Supplement: Supplementary file 5 — Supplementary figure legends [file 41419_2018_1216_MOESM5_ESM.docx]

**Supplementary Figure Legends**

**Fig. S1 Over-expression of FHC has no effects on ROS production**. Quantification of ROS production in A549^pc3DNA^, A549^pc3FHC^, H460^pc3DNA^ and H460^pc3FHC^ cells through the incubation with 20 μM of 2’-7’-DCF. Fluorescence was measured at 485 nm and 535 nm after 60 min. Results are the mean of three independent experiments performed in octuplicate.

**Fig. S2 Over-expression of FHC has no effects on NSCLC cell cycle progression**. Representative plots of cell cycle progression detected by FACS analysis in A549^pc3FHC^ *vs* A549^pc3DNA^ (**a**) and H460^pc3FHC^*vs* H460^pc3DNA^ (**b**) show no significant perturbations. FACS analysis was performed on three independent biological replicates. NS= Not Significant.

**Fig. S3 The pro-apoptotic effects of FHC over-expression do not affect the extrinsic apoptotic pathways.** Western blot analysis of three independent biological replicates of FAS and Caspase 8 protein levels in A549^pc3DNA^ *vs* A549^pc3FHC^ (**a**) and H460^pc3DNA^ *vs*H460^pc3FHC^ (**b**).

**Fig. S4 FHC over-expression affects miR-125b/p53 axis and promotes apoptosis in SW1573 and LXF-289 NSCLC cells.** TaqMan analysis of miR-125b expression levels (up) and Western blot analysis of FHC and p53 protein amounts (down) in SW1573^pc3DNA^ *vs* SW1573^pc3FHC^ and LXF-289^pc3DNA^ *vs* LXF-289^pc3FHC^. Each assay was performed on three independent biological replicates. **P*< 0.05 (**a**). Representative plots of Annexin V/ PI apoptosis assays in SW1573^pc3DNA^ *vs* SW1573^pc3FHC^ and LXF-289^pc3DNA^ *vs* LXF-289^pc3FHC^ (**b**). FACS analysis was performed on three independent biological replicates. *P*< 0.05.
